# Another Use for Carbolic Acid

**Published:** 1883-08

**Authors:** 


### ANOTHER USE FOR CARBOLIC ACID.

Some people suffer the most intense pain and annoyance from ingrowing toe nails. If the flesh has fully embedded the edges of the nail, and the tissue has become hypertrophied about it, cutting and paring seems but to aggravate the matter. When this is the case drop a very little pure carbolic acid along the borders of the inflamed tissue, and let it soak down beneath the nail. The pain will cease as if by magic, and the irritated flesh will soon make a healthy slough. If now the nail be scraped or filed very thin in the centre only, and from that back to its root, carefully leaving the edges alone, the growth will be directed toward the middle and a complete cure will result.

